# The Unconsidered Pathway: Suggestions for Physical Therapists to Facilitate Student Reintegration to Physical Education after a Concussion

**DOI:** 10.3390/children11101206

**Published:** 2024-09-30

**Authors:** Pamela Maree Tucker, Jennifer Strizak, Brian Rieger, Steven Lounsbury, John Leddy

**Affiliations:** 1 University of Pittsburgh Medical Center, Pittsburgh, PA 15219, USA; 2 Upstate Medical University Hospital, Syracuse, NY 13210, USA; strizakj@upstate.edu (J.S.); riegerb@upstate.edu (B.R.); lounsbst@upstate.edu (S.L.); 3 SUNY Buffalo Jacobs School of Medicine and Biomedical Sciences, Buffalo, NY 14203, USA; leddy@buffalo.edu

**Keywords:** concussion, mild traumatic brain injury, return to play, return to learn, physical education, physical therapy, pediatrics, adolescent, child

## Abstract

**Background/Objectives:** “Return-to-play” and “return-to-learn” after a concussion are familiar concepts due to guidelines proposed by the Concussion in Sport Group and Heads-Up Initiative. The purpose of this commentary is to expand upon the current consensus guidelines for treatment of concussed children and adolescents, as well as provide guidelines for returning to physical education (RT-PE) classes. **Proposal**: The authors propose one general and four subtype-specific guidelines post-concussion injury. This framework highlights the role of physical therapists in the management of children with prolonged recovery. The final RT-PE determination should occur with documented medical clearance from a licensed healthcare provider trained in the evaluation and management of a concussion. **Conclusions**: Despite significant gains regarding the management of concussed children and adolescents, confusion remains regarding RT-PE post-concussion. To eliminate ambiguity and promote adherence to a gradual return to activity protocols, the authors developed guidelines based on current evidence and recommendations.

## 1. Introduction

A concussion, a form of mild traumatic brain injury (mTBI), is a severe problem with increasing incidence in pediatric populations [1,2]. Defined as a complex pathophysiological process induced by biomechanical forces affecting the brain, concussion injuries cause an acute physiological disruption of brain function that typically does not have trauma-related abnormalities on standard structural neuroimaging studies [3,4]. The potential impact on the developing brain, including subtle changes in brain morphology and function, suggests the need for a safe return to physical activity (PA), including a return to physical education (RT-PE) guidelines [1,2,3,4,5,6,7,8,9,10].

Concussions accounted for more than 2 million outpatient visits and 3 million emergency department visits from 2005 to 2009 in the United States, though research indicates that more than 50% of concussions are likely to go unreported to healthcare professionals [1,2,8]. Adolescents aged 13 to 19 are at the highest risk due to the rising number of student–athletes involved in sports activities [9,10]. While organized sports have the highest incidence of a concussion, data from 1408 pediatric patients at Saint Peter’s Sports Medicine Institute from September 2014 to May 2018 revealed that the second highest incidence of a concussion was from physical education (PE) classes [11]. Although most cases resolve within weeks, at one-month post-injury, nearly a quarter of children report a headache, over 20% suffer from fatigue, and nearly 20% report increased processing time [12]. These symptoms can adversely affect school participation, PA, and quality of life [13,14,15].

## 2. Clinical Question

What recommendations exist in the literature regarding a safe return to PE class after a concussion for school-aged children and given the current level of evidence, how can physical therapists facilitate the transition of concussed children and adolescents back to PE class?

## 3. Background: Return to Physical Education Is Not the Same as Return to Play

In 2001, the multidisciplinary Concussion in Sport Group (CISG) of sport and medical professionals convened the First International Conference on Concussion in Sport (ICCS) with the objective of improving the evaluation, management, and return-to-play (RTP) of concussed athletes [16]. At the fifth meeting in 2016, the ICCS recommended that student–athletes should return to school prior to returning to sport and noted that the early introduction of symptom-limited PA is appropriate [17]. These suggestions are consistent with educational initiatives, such as the Centers for Disease Control Heads-Up program, to facilitate return-to-school after a concussion and support gradual re-entry with accommodations that taper as the student’s symptoms improve [16,17,18,19,20,21]. Current return-to-school recommendations, however, focus primarily on the management of environmental stimulation and cognitive demands with little guidance on returning to PA despite recent updates by the New York State Education Department to its Concussion Management Guidelines for Schools to better differentiate between a return to athletic activity, which includes after-school sports, and a return to physical activity, which includes PE class [15,16,17,18,19,20,21,22,23,24,25,26].

Tsuchida and colleagues recently outlined a RT-PE progression following the standard scholastic sports RTP guidelines. However, this program does not begin until a student is medically cleared from a concussion [27]. This is the most common approach followed in schools, and many physicians will provide a note indicating that a student is “out of sports and PE until cleared”. Whether a student recovers quickly from a concussion or experiences a more prolonged course, it is appropriate for the student to re-engage in the PE curriculum whenever they are back in school, even if still symptomatic. Just as adjustments or accommodations are made in other academic subjects, modifications to assignments and in-class participation can allow PE teachers to meet individual students’ needs. While there is understandable concern about the risk of re-injury associated with PA, there is evidence that symptomatic student–athletes who engage in light PA recover faster from a concussion with noted psychological benefit [13,28,29,30,31,32,33,34,35,36,37,38,39]. Furthermore, the most recent recommendations from the CISG (Amsterdam 2022) specifically note that mild symptom exacerbation (i.e., no more than a 2-point increase in concussion symptoms on a 0–10 scale when compared with the pre-activity level) during physical or cognitive activity is typically brief (less than one hour), is not harmful, and does not delay recovery [3,40].

In youth who sustained a sport-related concussion (SRC), Leddy and colleagues demonstrated that participation in light PA within 48–72 h of injury, beginning at approximately 50–60% of the age-appropriate maximum heart rate, safely facilitated recovery when compared with stretching-exercise controls [34,35]. The psychological consequences of removal from validating life activities combined with physical deconditioning may contribute to the development of persisting symptoms after mTBI [10,11,12,29,31,32,33,39]. Given the emerging evidence, we suggest that it is safe to allow symptomatic students to progress through a guided return to a physical education (RT-PE) program with supervision. Additionally, we propose that physical therapists can play an essential role in facilitating RT-PE in instances of prolonged (>10 days of persistent symptoms) or complex (multi-symptom or severe symptoms) injury, and their observations can provide the academic team with important information regarding readiness of the student to advance along the continuum of graduated RT-PE.

The RTP protocol for sports seeks to establish clinical recovery over the course of an increasing exertional and coordination challenge under the supervision of a qualified healthcare practitioner [7,8]. Per the 2022 Amsterdam guidelines, this graduated stepwise strategy occurs in conjunction with return-to-learn (RTL) following an initial period of relative rest (approximately 24–48 h following initial injury), though participation in activities of daily living including walking is permitted immediately following injury [3,4,5,24,25,41]. RTL programs encourage student participation in symptom-limited academic effort (Table 1).

The authors acknowledge that PE class involves more physical exertion than subjects such as Math or English, but emerging evidence documents a protective effect of active rehabilitative interventions such as PA in concussion management [33,42,43,44,45,46]. A 2016 pilot study by Gagnon and colleagues found that light PA in the post-acute period following a concussion is safe and yields a positive impact on adolescents’ functioning [31]. Furthermore, a multicenter cohort study in 2016 by Grool et al. of 3063 children aged 5–18 years noted that the proportion with post-concussive symptoms at 28 days was 28.7% with participation in early PA versus 40.1% with conservative rest [33]. This corroborates previous randomized control studies by Leddy and colleagues from 2017, 2019, and 2021, which found strict physical rest until symptom resolution to be an ineffective method to treat a concussion in adolescents [34,35,36].

Although school districts generally do not require concussion management training for physical educators, secondary responsibilities such as coaching drive many to pursue online training [47]. While this training yields a greater understanding of concussion policies and procedures, confusion remains regarding implementation in PE class [22,41,47]. In the authors’ experience, many schools do not allow participation in PE until the student has completed the RTP protocol and is cleared to return to contact sports. Rather than participating in regular PE activities, students complete stationary cognitive work—even though doing so may aggravate symptoms. Schools may require make-up PE classes once students recover, while others are relegated to sitting in the gymnasium to watch classmates participate, despite the potential for symptom exacerbation. Such restriction from class participation may cause additional stress and contribute to the student feeling abnormal [8,46,48]. To address this problem and encourage greater consistency, we propose a stepwise progression for RT-PE that occurs in parallel with the current RTL and RTP progressions and supports students not engaged in school-sponsored athletics at the time of injury. These guidelines were developed to best support typically developing school-aged children (approximately 3–18 years of age) with and without access to structured school-sponsored athletics to recover safely post-concussive injury.

## 4. Description of Topic

The following RT-PE guideline was developed after an extensive literature review of five databases (PubMed, Cochrane library, PEDro, CINAHL, and Scopus) revealed no specific protocol for a return to PE after concussion injury. These guidelines were created based on the summation of evidence-based recommendations from this search, in addition to the collaborative efforts of a multidisciplinary medical team including physicians, psychiatrists, and therapists specialized in concussion rehabilitation at both the SUNY Upstate Concussion Center and the SUNY Buffalo Jacobs School of Medicine and Biomedical Sciences. These suggestions were reviewed and supported by the Brain Injury Association of New York State (BIANYS), as well as expert opinion from physical educators, school administrators, school-based therapists, school-based athletic trainers, and other multidisciplinary concussion-specialized medical professionals across the states of New York, Connecticut, and Texas. These guidelines are currently being implemented in association with three upstate New York school districts over the course of one academic year (2024–2025) and expand on recent recommendations from Rieger and colleagues for physical educators and school administrators [49], to propose suggestions for physical therapists to facilitate safe RT-PE for students affected by a concussion (Table 2).

**Figure 1:**
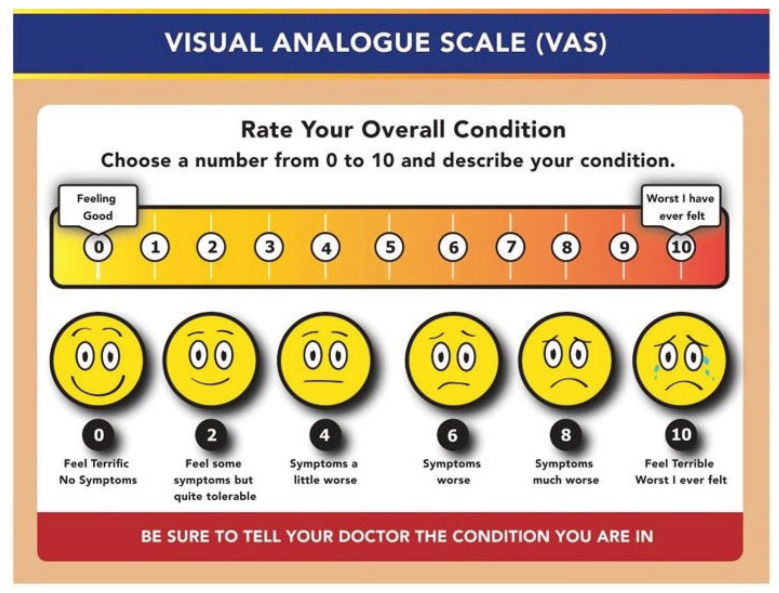
Visual Analog Scale adapted from Hayes and Patterson [50].

These guidelines aim to maintain the integrity of the current six-step RTP and the four-step RTL strategies suggested by the ICCS [3,24,25,26]. The authors propose this general RT-PE guideline that can be implemented after injury with agreement and supervision from both the medical and academic teams. To this end, Leddy and colleagues found the Visual Analog Scale (VAS) to be an easy and effective tool for assessing overall symptom burden (Figure 1) [34,35,50,53].

They recommend the initiation of light PA if symptom severity at rest is <7/10, and stopping PA when symptoms rise by more than 2 points on a 0–10 scale when compared with the pre-activity resting value [34,35,36].

The authors further propose four subtype-specific RT-PE protocols for patients who may experience a more complicated recovery (Appendix A), as described by the ICCS as experiencing persisting neck pain and/or headache for more than 10 days or symptoms, such as dizziness or balance problems, persisting beyond 4 weeks across all age groups [3,16,17,18,19,20,21]. These follow the Concussion Clinical Practice Guideline (CPG) Classifications of impairments in the domains of cervical musculoskeletal, vestibulo-oculomotor, autonomic dysfunction and exertion intolerance, and motor function [54]. These recommendations are not strict protocols, but are suggestions based on the current evidence of best practice for concussion management. They will benefit from empirical analyses and assessment of cost effectiveness and resource utilization as the pilot program unfolds through the Upstate Medical University hospital system and participating New York state school districts over one year (2024–2025).

## 5. Proposed Implementation

**Figure 2:**
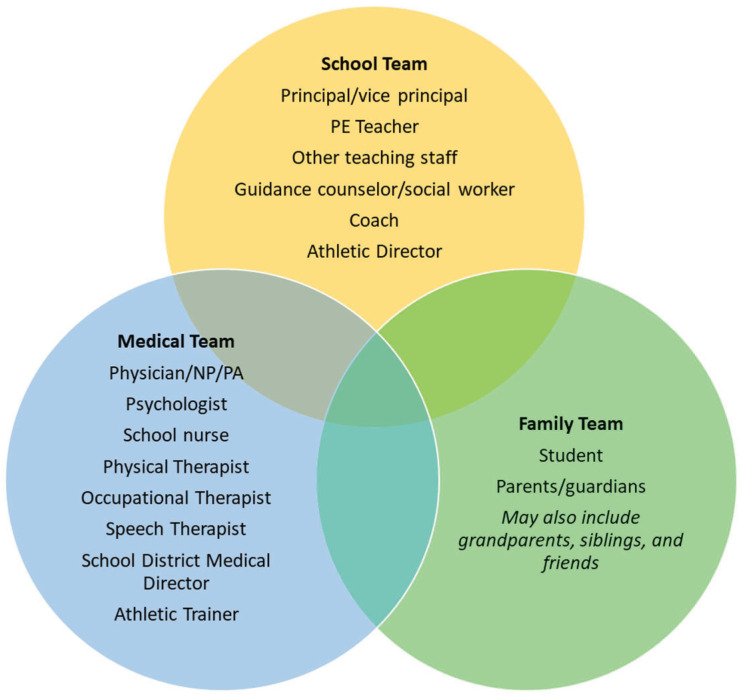
A multidisciplinary approach to concussion management adapted from the Brain Injury Association of New York State and the Colorado Department of Education’s Reduce, Educate, Accommodate, Pace (REAP) program [6].

After a concussion, the student engages with healthcare professionals who provide appropriate management, including recommendations related to school (Figure 2) [7].

The provider who completes the initial assessment determines immediate activity restrictions and clearance to attend school. First-line providers should educate the family about concussion risks and document all recommendations in writing for the family, school, and other medical team members. Barriers identified to concussion management and family education include inadequate training, time, and infrastructure to successfully manage these patients [55]. Where the clinical environment allows, we recommend that the student be referred to clinicians with specialized knowledge and skills in concussion management for further evaluation and targeted treatment of symptoms. After assessment, an appropriate medical professional determines cognitive activity restrictions [56]. A medical provider educated and experienced in concussion management and physical training progression, such as a physical therapist, may provide PA recommendations including when RT-PE may be attempted [57]. Individualized accommodations should be provided in writing to both the family and the academic team [6,34,56,57]. We include a sample letter that physical therapists can complete quickly in the clinical setting (Supplementary Material File S1) to increase the likelihood that they will receive support from their school [6,41]. As the academic team is required to facilitate the return for all students with a concussion, regardless of athletic status or access to athletic trainers, we propose that PE teachers fill this role as a direct contact for the medical team to assess the student’s tolerance to PA (Figure 2). Open, regular, and clear communication between the various teams throughout the program will promote its overall success.

Many states have passed legislation designed to address the growing concern of concussions among students. The Colorado Department of Education’s Reduce, Educate, Accommodate, Pace (REAP) program is a community-based concussion management approach that has been adapted across several states with the goal of creating a wide safety net for students as they recover from a concussion [6]. Our protocol builds on the multidisciplinary model proposed in REAP, as well as the Return to Activity Guidelines for Concussion and Youth proposed by DeMatteo and colleagues in 2014 and subsequentially implemented across Ontario [1,2]. Unique aspects of the Canadian protocol include an emphasis on conservative management, striving to balance brain healing and active participation, and multiple pathways to promote recovery [2]. Our protocol is currently being piloted at the Upstate Concussion Clinic in Syracuse, NY, and in New York state school districts in collaboration with BIANYS and local athletic trainers. Though sound in model, a quantitative analysis as to the efficacy and cost effectiveness of this program cannot be established until formal data collection occurs after the completion of the one-year trial, which the authors recognize as a limitation.

Students with typical uncomplicated presentations should follow the general RT-PE protocol outlined in Table 2 as tolerated, starting at Step 1 immediately and for the first 24–48 h post-injury. Students with more complex presentations such as multiple, severe, or persisting symptoms as identified by the ICCS may require further assessment to manage their recovery [3,16,17,18,19,20,21,26,54]. Progression through both guidelines is based on overall tolerance and symptomology, as well as performance in the various activities at each stage. Like the RTP guidelines, each stage lasts a minimum period of 24 h. Due to the lack of a specific reconditioning program post-concussion, our six-step programs must be individualized to fit each student’s presentation. Proper form and technique should be emphasized, and intermittent rest breaks should be provided to avoid symptom exacerbation. Per the Concussion CPG, the guiding criterion for the progression of aerobic exercise is the reduction in symptom irritability [54]. In their 2018 study, Lawrence et al. utilized a standardized stationary bike protocol with progressive time and intensity intervals [58]. The researchers recommend a minimum of two sessions tolerated at each level prior to progression to the next stage. While these recommendations provide a foundation for our proposed progression of aerobic activity, we suggest the use of a modified Borg rating of the perceived exertion (RPE) scale to monitor the student’s activity level if heart rate monitoring equipment is not readily available [51].

When reintroducing resistance training, the student’s one-repetition maximum (1RM) can be estimated using a 10RM load following established guidelines [59]. The student should start at a lower relative intensity (20% 1RM) at 10 repetitions with gradual progression to higher repetitions (12–20) to improve muscular endurance [59,60]. As endurance improves, the relative intensity increases to a moderate level (50% 1RM) with 8–12 repetitions to build muscle strength. In appropriate student–athletes, this should then progress to a strength–power focus (85% 1RM) with only 2–8 repetitions that mimic more sport-related movement patterns. The progression of the resistance training program should be based on the student’s ability to complete the exercise with good form without symptom exacerbation [59,60,61].

The proposed PE class activities in our guidelines support the product (fitness) and process (PA and other healthy lifestyles) proposed by the Society of Health and Physical Educators (SHAPE) in their Instructional Framework for Fitness Education in Physical Education and align with the National Standards for K-12 Physical Education (2013) and accompanying Grade-Level Outcomes (2013) [62]. The framework suggests what students should understand and be able to carry out at specific grade levels, focusing on helping them adopt a healthy lifestyle during the years of education ranging from prekindergarten to college. Our guidelines build upon this curriculum framework and gradually progress the students to more complex coordination and motor planning activities to facilitate reintegration into their premorbid school and PA environment.

## 6. Subtype-Specific Guidelines

While more than 80% of pediatric concussions resolve within three weeks, a subset of patients experience prolonged symptoms that can persist for months to years and require more specialized outpatient management [4,9,10,63,64,65,66]. The timeline of recovery may vary from patient to patient based on factors such as history of previous concussions, severity of initial symptoms, age, and gender [7,8,11,65]. As multiple studies report a risk of re-injury most notably for pediatric patients within the first 10 days of injury [8,16,17,18,19,20,21,32,44,56,63,65], physical therapists can play an essential role in the multidisciplinary team. Therapists allow patients to achieve a controlled level of exercise and stimulation, offering patients a more fulfilling and safer lifestyle during recovery [57]. Physical therapists are uniquely positioned to evaluate the various impairments students may exhibit post-concussion and to classify a specific treatment track for recovery as outlined in the Concussion CPG from the American Physical Therapy Association [54].

Once referred for examination, we recommend that therapists screen and clear patients for signs of a medical emergency or severe pathology and then complete a comprehensive evaluation identifying relevant impairments in the domains of cervical musculoskeletal function, vestibulo-oculomotor function, autonomic dysfunction/exertional tolerance, and motor function through foundational standard-of-care screening strategies [54]. Provocative testing of neurocognitive function, exercise tolerance, and vestibular-oculomotor function are important components of this evaluation, as symptoms and performance may be normal in the resting state [67]. Although we acknowledge that many patients will present with symptoms that may encompass multiple tracks, we propose that therapists identify the patient’s primary limitations and utilize the most appropriate subtype-specific guideline to facilitate recovery. Clinicians are strongly encouraged to monitor the student throughout their rehabilitation, with feedback from the academic and family teams, to determine whether the student remains appropriate for the chosen rehabilitation track or if updates to the plan of care are necessary.

Although our recommendations are based on the available literature, we recognize that the current body of evidence from which to draw conclusions is limited. The Concussion CPG is new, and the four domains were recently established. Therefore, an optimal battery of assessments and interventions following standardized protocols addressing the various aspects of recovery does not currently exist. We further recognize that the stages and interventions for our described PE activities have yet to be validated for PE of physical therapy in a concussion cohort. However, therapists can complete developmental functional testing of motor and postural control, perceptual and oculomotor abilities, and screening of the vestibulo-ocular and vestibulo-spinal systems to establish whether functional balance and/or oculomotor deficits exist, isolate the contributions of the various components of postural control and gaze stability, and provide a basis for referral for further diagnostic testing [68,69]. Students scoring below age-appropriate levels identified in screening for vestibulo-ocular or vestibulo-spinal dysfunction should be referred for more comprehensive testing (e.g., oculomotor, caloric, vestibular evoked myogenic potential (VEMP) testing). Therapy interventions should consider the inclusion of balance, adaptation, and substitution training modified to the child’s level of cognitive maturation and interest level, with consideration for the caregiver assisting with the activities in the home exercise program. Our suggested interventions and stages draw from general guidelines for exercise prescription and progression by Herdman et al. [70], as well as tested precision vestibular rehabilitation programs [71,72,73,74,75]. Current evidence supports this multi-modal approach to address individual impairments including modalities, manual therapy, and therapeutic exercise [38,72]. The authors propose that physical therapists utilize the suggested guidelines to promote habituation and neurological compensation following three principal methods of exercise: gaze stabilization, habituation, and balance/exertional training [70,71,74,75,76,77]. Finally, as many vestibular programs draw from unilateral hypofunction, which has an undetermined frequency in concussion injury, we encourage therapists to consider the inclusion of sensory integration or delayed motor responsiveness interventions to the plan of care as appropriate.

A customized problem-oriented approach is the standard intervention for rehabilitation post-concussion [43,54,56,57,64,70,72,78]. The general rationale for exercise prescription in the subtype-specific protocols was developed using the following: the (1) identification of the impairments during evaluation, (2) prescription of a specific initial exercise to address the impairments safely, and (3) progression of the exercise to include functional performance at increasing levels of difficulty. For instance, post-concussion vestibular impairment frequently presents functionally as balance dysfunction, visual motion sensitivity, vestibulo-ocular reflex (VOR) impairment, and/or post-traumatic benign paroxysmal positional vertigo (BPPV). The implementation of vestibular rehabilitation is recommended when deficits are identified in any of these areas [67]. The VORx1 exercise may be used as an example. Students may start in static, stable positions such as standing with feet apart or sitting. This activity may progress to increasing the VOR gain demand by increasing head velocity, decreasing the distance to the target, varying target size and complexity, or switching to a VOR x 2 paradigm [73]. Additional balance progressions may be considered such as standing with feet together, standing tandem, and eventually walking [72]. Because vestibular interventions, most notably treatment of visual motion sensitivity, have the potential to exacerbate symptoms, interventions should be introduced in a step-by-step progression that is carefully monitored by a trained vestibular therapist [67,70,73,79].

The same principles were utilized when selecting recommendations for each subtype domain. As seen in the musculoskeletal domain, therapeutic exercise interventions should start by addressing the identified impairments [57,78,79,80]. For instance, a combined approach of mobilization techniques and postural awareness training may facilitate symptom management [81,82]. Reinforcement of appropriate biomechanics with activity can restore muscle balance and affect motor control deficits. Exercises to improve upper quarter strength may demonstrate a positive impact on symptom management for cervicogenic dysfunction [78]. We propose that therapists develop an individualized exercise program based on findings from the assessment, which may then be implemented during PE class in place of or in conjunction with planned activities as tolerated by the student. Therapists are encouraged to remain in frequent communication with the various teams, most notably the referring physician and the PE teacher, regarding the student’s progress through the various stages of recovery to facilitate the eventual discharge from the program and clearance for pre-injury-level activity.

## 7. Conclusions and Future Directions

Current guidelines for rehabilitation after concussion injury fail to describe how students may return to PE class, which potentially extends their recovery timeline and limits their participation in validating activities. The authors propose progressive six-step programs, which may be utilized by healthcare, academic, and family team members to facilitate the student’s RT-PE. Successful concussion management occurs if the student can maintain their pre-injury academic progress without lasting adjustments and safely return to activity, minimizing the potential for social isolation and depression/anxiety symptoms. These guidelines bridge the gap between the current RTL and RTP protocols and promote the rehabilitation of both athlete and non-athlete students. Although some research suggests that prolonged physical and cognitive rest may be beneficial to recovery [83], the evidence overwhelmingly supports an early and gradual return to activity to maximize overall rehabilitation [3,40,42]. We recommend that each case be implemented on an individual basis and students be monitored continuously for progress and any potential worsening of symptoms. These recommendations provide a framework to allow clinicians and schools to communicate more openly about the student’s overall progress, limitations, and support needs.

As these guidelines are currently being implemented on a trial basis through Upstate Medical University and surrounding New York school districts, a significant limitation of these guidelines is that they do not currently have empirical evidence to support their use. Further research is necessary to validate their potential impact on RTP and RTL efficacy, their cost effectiveness and resource management, and their overall success. Additionally, given the heterogenous nature of concussion injuries, the authors emphasize that the universal prescription of any intervention, including the suggested guidelines, to all concussed students is an inefficient strategy. Instead, comprehensive scholastic and clinical assessments, the targeted implementation of accommodations and interventions, and shared communication between the various teams are supported.

A practical barrier to the implementation of these guidelines is the knowledge and expertise to guide students through these guidelines effectively. We highly recommend frequent and clear communication between the academic, family, and medical teams to ensure the appropriate implementation of these suggestions. Finally, variations in resources across school districts will necessitate the adaptation of the suggested guidelines based on availability. Although these guidelines may potentially benefit students outside of the United States educational system, the authors acknowledge that further updates may be necessary based on the educational program, resources, and culture specific to the student’s environment. As there is limited empirical support for the rehabilitation strategies discussed in these guidelines, the authors suggest future research to include multi-site, randomized controlled trial research designed to better elucidate the specific effects of these interventions and strategies.

Despite these limitations, the authors propose that these guidelines serve as a stepping stone to support students who would be otherwise under-supported by the current protocols. The hope is that these guidelines serve as a living document that may enhance and support various programs across the United States and world educational communities, with adaptations and updates as necessary given future insights and resources.

## Figures and Tables

**Table 1:** Return to Play Versus Return to Physical Education.

Return to Play	Return to Physical Education
Begins when student is concussion symptom-free	Begins when student can tolerate being in school for at least part-time, even if still symptomatic
Goal is to verify recovery for unrestricted return to sport	Goal is to help the student participate in normal school activities during recovery
The same protocol is used for every student–athlete	Every student is affected differently and needs accommodations tailored to fit
Uniform protocol well known to athletes, parents, coaches, and others	Many schools and teachers remain unsure how to manage this because there are no uniform guidelines
Mostly overseen by athletic trainer and/or school nurse	Requires teachers and administrators working closely with medical team and student/family

**Table 2:** RT-PE General Guideline.

Step	Status	Return to PE Progression	Other Information
1	Period of relative rest occurring immediately and for up to the first 2 days after injury Symptoms are acute, experiencing symptoms at rest and/or symptom onset immediately with activity Student not at school	Activities permitted at home: - Slow walking for short durations, stopping if symptoms increase by >2 points on 0–10 VAS [50] - Daily tasks and chores (dishes, meal prep, light cleaning) - Movements that can be performed with little effort (do not increase breathing and/or heart rate significantly) Student moves to Step 2 when able to complete activity described above with “mild” (no greater than 2-point increase) and “brief” (<1 h) symptoms and no new symptoms	Activities to avoid: - Physical exertion that elicits more-than-mild symptom exacerbation - Sports activity - Stair climbing other than to move locations throughout the home - Activities that require mental stimulation or concentration such as computers, excessive phone use, or videogames - High environmental complexity (i.e., loud music or bright lights) - Roughhousing or activities with increased risk of falls - Driving (until cleared by MD)
2	May be symptomatic or asymptomatic at rest, symptomatic with activity Student has returned to school	Activities permitted in PE class: - Participating in a progressive walking program or stationary bike propulsion in a quiet environment (if possible) o Duration determined by symptom threshold-terminate activity if symptoms increase by >2 points on 0–10 VAS from pre-activity level [50] - Light-intensity yoga and/or stretching when sitting or standing without inversion	- Exertion testing may be completed by PT or ATC such as the Buffalo Concussion Treadmill Test (BCTT) [37] - Student should not be in loud or brightly lit space if possible - Mild aerobic exercise (pace that causes some increase in breathing/heart rate but not enough to prevent carrying on a conversation comfortably; modified Borg Rate of Perceived Exertion (RPE) 2–3/10 [51] Activities to Avoid: - Resistance training - Risk for contact/collision - Activities that rapidly increase heart rate
3	Symptoms do not increase by more than 2 points with activity in Step 2 Student may be asymptomatic or symptomatic at rest	Activities permitted in PE class: - Aerobic exercise: stationary bike, elliptical, and/or treadmill or flat-surface jogging for progressive intervals with walking “breaks” in between - Initiate simple manipulative activities as tolerated (e.g., ball pass/catch, dribbling, etc.) - Individual activities in gym (e.g., throwing drills, shooting drills in basketball and soccer) in predictable and controlled environments with no risk of re-injury - Initiate bodyweight and isotonic weight training (e.g., bodyweight squats and pushups, 1 set of 10 reps each)	- Moderate aerobic activity (modified Borg RPE 4–6/10) [51] - Bodyweight/light resistance training (approximately 25% of pre-injury 1 rep max if known), or may utilize RPE 4/10 [51] - Restricted recess activities (individual play only) Activities to Avoid: - Heavy/high-weight resistance training - Risk for contact/collision - Activities that rapidly increase heart rate - Jarring motions such as high jumps and lands, inversion movements, or plyometrics
4	Symptoms improving, occurring less often and at lower intensity, symptoms do not increase more than 2 points with activity in Step 3 Student may be asymptomatic or symptomatic at rest	Activities permitted in PE class: - Progressively increase physical activity to non-contact training drills to add coordination and increased thinking challenges - Initiate simple locomotor activities (galloping, hopping, leaping, skipping) - Dynamic warmups (high knees, butt kicks, carioca, etc.) - Unit-specific non-contact activity in a SAFE environment modified for individual work - Middle school/high school: Progress resistance program to circuits (e.g., squat, deadlift, bench press, etc.) - Aerobic exercise within gym environment and/or outdoors	- Moderate aerobic activity (modified Borg RPE 4–6/10) [51] - Bodyweight and moderate resistance training (up to 50% pre-injury 1 rep max) or may utilize modified Borg RPE 5–6/10 [51] No risk for contact/collision Recess—physical activity running/games with no body contact. Limit use of playground equipment that places the child in an elevated position, such as swings and monkey bars
5	No increase in symptoms with activities performed in Step 4	Activities permitted in PE class: - Unit-specific non-contact activity relevant to curriculum benchmarks in a SAFE environment o Initiate small-group skill work: station work, small side volley games, more intense aerobic activity - Progress training drill complexity gradually with or without a partner (e.g., passing drills in soccer and hockey, post moves and rebounding in basketball, fielding ground balls and throwing/catching in baseball) - Initiate participation in practices for non-contact interschool sports Elementary: throw and catch with a partner using safe equipment, rhythm activity, station work Middle school/high school: high-resistance weight training with a spotter - Plyometric exercises (burpees, squat thrusts, mountain climbers)	- Moderate to high aerobic activity (modified Borg RPE 7–8/10) [51] - Bodyweight and moderate resistance training (up to 85% pre-injury 1 rep max) or use of RPE 7–8/10 [51] No risk for contact/collision
6	Return to all activities—transition to RTP protocol as needed		If participating in a physical therapy plan of care, the student scores within normal or expected ranges per balance, exertion, and symptom assessment measurements as indicated per the nature of their illness and impairments. Consider completion of return-to-sports assessment (e.g., Dynamic Exertion Test for Concussion) [52].

## Supplementary Materials

The following supporting information can be downloaded at: https://www.mdpi.com/article/10.3390/children11101206/s1, File S1: Sample School Accommodation Form.

## Abbreviations

MCID	Minimum Clinically Important Difference
ATC	Certified Athletic Trainer
MD	Medical Doctor
E	Elementary School
MS	Middle School
HS	High School
HR	Heart Rate
RPE	Rate of Perceived Exertion
AROM	Active Range of Motion
PROM	Passive Range of Motion
UE	Upper Extremity
SOT	Sensory Organization Test
BOT-2	Bruininks–Oseretsky Test of Motor Proficiency Edition 2
CTSIB-M	Modified Clinical Test of Sensory Interaction in Balance
Bess	Balance Error Scoring System
PDMS-2	Peabody Developmental Motor Scale Second Edition
HIIT	High-Intensity Interval Training
DHI	Dizziness Handicap Inventory
DGI	Dynamic Gait Index
POTS	Postural Orthostatic Tachycardia Syndrome
FGA	Functional Gait Assessment

## Appendix A. Return to Physical Education Subtype-Specific Guidelines

- RTL and RT-PE are interrelated, but not interdependent.
- Progress through RTL is independent of progress through RT-PE.
- Each step must last a minimum of 24 h.
- Step 1 occurs at home; Steps 2 and thereafter occur at school.
- The student progresses to the next step when able to complete all activities of the current step with no greater than mild (two-point increase in symptoms per the VAS) (Figure 1) [50] and brief (<1 h) symptom exacerbation, and no new symptoms.
- If the student experiences symptom exacerbation >2 points per the VAS scale (Figure 1) [50], they are advised to stop the triggering activity for a therapeutic break. If symptoms improve, they may return to that activity [40].

Initial Relative Rest

- The student begins at Step 1 immediately and for up to 24–48 h post-injury [40,44,56].
- The student moves to Step 2 when symptoms begin to improve or after 48 h (whichever occurs first) [40,44].

**** The student must return to previous step for a minimum of 24 h when they exhibit or report more than a mild (>2 points on a 0–10 scale) or brief (>1 h) exacerbation of current symptoms or new symptoms evolve.

**** The student should see a medical doctor if symptoms do not improve by the following day or if new symptoms arise and persist.

**** The student may begin in one subtype guideline, though their progress should be monitored throughout by a skilled clinical professional as students may cross-over to a different guideline as they progress through their rehabilitation.

**** If progression through these guidelines is limited by factors outside of concussion injury, the authors suggest progression through the activities as directed by the medical team to the best of the student’s abilities to facilitate the long-term goal of attaining pre-injury mobility status.

**Table A1:** Cervical Musculoskeletal Impairment Guideline.

Step	Status	Return to PE Progression	Physical Therapy Program
1	Symptoms are acute at rest and/or onset immediately with activity Potential student presentation: - Headaches and/or neck pain - Reported visual difficulties including challenges with focusing on targets - Impaired balance and/or dizziness - Painful or limited cervical AROM - Impaired Cervical Joint Position Sense (error or decreased control) [84] - Decreased motor control in deep neck flexors and/or extensors	Activities permitted at home: - Slow walking for short durations, stopping if symptoms increase by >2 points on 0–10 VAS [50] - Daily tasks and chores (dishes, meal prep, light cleaning) - Movements that can be performed with little effort (do not increase breathing and/or heart rate significantly) Student moves to Step 2 when able to complete activities described above with no more than “mild” (no greater than 2-point increase) and “brief” (<1 h) symptoms and no new symptoms	Physical therapy assessment may include: - Patient-reported outcome measures (e.g., Post-Concussion Symptom Scale, Rivermead Questionnaire, Neck Disability Index, Headache Disability Index, Concussion Clinical Profiles Screen) [84,85,86,87,88] - Cervical and thoracic mobility/ROM [57,68,69,78] - Cranial nerve exam [57,72] - UE myotomes, dermatomes, and reflexes [57,68,69] - Palpation of cervical and scapulothoracic muscles [57] - Special tests including, but not limited to, cervical ligament stability, smooth pursuit neck torsion test, cervical flexion rotation test, head–neck differentiation test, postural stability testing, cervical torsion test, and cervical joint position error test for cervical proprioception [68,69,79,89,90] - Motor control assessments of deep neck flexors and extensors [68,80] - Exertion assessment as appropriate [37,57,67,91]
2	Symptomatic or asymptomatic at rest Symptomatic with activity Student has returned to school (for reduced time or with restrictions) Potential student presentation: - Neck pain may remain present at rest - AROM may remain limited and/or painful - May report difficulty with motor control/feelings of heaviness - Reported “fogginess”	Activities permitted in PE class: - Participating in a progressive walking program or stationary bike propulsion in a quiet environment (if possible) - Duration determined by >2-point increase in symptoms on 0–10 scale from pre-activity level - Light-intensity yoga and/or stretching when sitting or standing without inversion - Cervical AROM exercises within range that does not increase pain - Standing upper-quarter exercises such as banded rows, shoulder extension, bicep curls, and tricep extension	Physical therapy treatment may include: - Low-load cervical-specific exercises (as indicated) for ROM and pain modulation - Suboccipital/upper trapezius/levator scapulae stretching - Cervical isometrics in specific directions - Craniocervical flexion/extension endurance - Postural exercises [68] - Cervical proprioception exercises in siting, sensorimotor re-training [68] - Progressive isometric holds of motor control exercises [73,79] Goals to progress to next step: - Improving cervical AROM and PROM - Decreasing cervical pain levels on VAS [50] - Joint position error improving or moving towards normal (<4.5 degrees/7.1 cm) with improved quality of movement if indicated [80,81,82,90]
3	Student may be asymptomatic or symptomatic at rest Symptoms do not increase by more than 2 points with activities performed in Step 2 Potential student presentation: - No neck pain at rest, though this may be reproduced by cervical AROM - Normal or nearing normal cervical AROM and PROM	Activities permitted in PE class: - Aerobic exercise: stationary bike, elliptical, treadmill, or flat-surface jogging for progressive intervals with walking “breaks” between - Initiate simple manipulative activities as tolerated (i.e., ball pass/catch, dribbling, etc.) - Individual activities in gym (e.g., throwing drills, shooting drills in basketball and soccer) in predictable and controlled environments with no risk of re-injury - Initiate bodyweight and isotonic weight training (e.g., bodyweight squats and pushups, 1 set of 10 reps each) [59] - Low-weight, 10–15 repetitions of upper-quarter dumbbell exercises [59]	Physical therapy treatment may include: - Progression of cervical and postural strengthening - Use of free weights for prone/inclined/standing postural exercises (rows, ITY, etc.) - Cervical proprioception exercises in standing - Deep neck flexor and/or extensor endurance exercises [92] Goals to progress to next step: - Achieving norms for deep neck flexor endurance test (e.g., 38.9 s for men and 29.4 s for women) [92] - MCID for appropriate outcome measures (e.g., Headache Disability Index, Neck Index) [84,86]
4	Symptoms improving, occurring less often and at lower intensity, symptoms do not increase with activity in Step 3 Potential student presentation: - Asymptomatic or symptomatic at rest	Activities permitted in PE class: - Progressively increase physical activity to non-contact training drills to add coordination and increased thinking challenges. - Initiate simple locomotor activities (galloping, hopping, leaping, skipping) - Dynamic warmups (high knees, butt kicks, carioca, etc.) - Unit-specific non-contact activity in a SAFE environment modified for individual work - Aerobic exercise may be in the gym environment and/or outdoors - Progress resistance program to circuits (e.g., squat, deadlift, bench press, etc.) [59]	Physical therapy treatment may include: - Cervical proprioception exercises in standing, improving accuracy with less symptoms [68,79] Cervical-specific exercises: - Continue to progress cervical and postural strengthening with and without resistance - Progress resistance prone/inclined/standing postural exercises (rows, ITY, etc.) Goals to progress to next step: - Full cervical AROM without pain - Normal or near-normal motor control (deep neck flexor and deep neck extensor) tests [45,64,79]
5	No increase in symptoms with activities performed	Activities permitted in PE class: - Unit-specific non-contact activity relevant to curriculum benchmarks in a SAFE environment - Initiate small-group skill work: station work, small side volley games, more intense aerobic activity - Progress gradually to complex training drills with or without a partner (e.g., passing drills in soccer and hockey, post moves and rebounding in basketball, fielding ground balls and throwing/catching in baseball) - Participation in practices for non-contact interschool sports Elementary: throw and catch with a partner using safe equipment, rhythm activity, station work MS/HS: - High-resistance weight training with a spotter - Plyometric exercises (burpees, squat thrusts, mountain climbers)	Physical therapy treatment may include: - Integrate sport-specific movements and activities into postural and cervical exercises (e.g., review and progress header form and safety as appropriate for soccer players, add acceleration/rotational forces in a controlled setting for gymnasts and cheerleaders, address technique with hit/push pad then sleds with football players, etc.) Goals to progress to next step: - Continued pain-free cervical AROM - Normal craniocervical flexion and deep neck flexor endurance, no pain during these tasks - Tolerating standing cervical proprioception exercises with no increase in symptoms [79,93,94]
6	Discharge from PT—transition to RTP protocol as needed		The student scores within normal or expected ranges per balance, exertion, and symptom assessment measurements as indicated per the nature of their illness and impairments. Consider completion of return-to-sports assessment (e.g., Dynamic Exertion Test for Concussion) [52,94]

**Table A2:** Vestibulo-oculomotor Impairment Guideline.

Step	Status	Return to PE Progression	Physical Therapy Program
1	Symptoms are acute, experiencing symptoms at rest and/or symptom onset immediately with activity Potential student presentation: - Headache - Dizziness and/or vertigo - Nausea - Balance problems and difficulty focusing on objects (stable or unstable) and reading - Screen intolerance - Visual disturbance (blurred vision or motion sensitivity) - Abnormal extraocular movements - Abnormal or symptomatic saccades or smooth pursuits - Abnormal Head Impulse Test - Near Point of Convergence >6cm - Abnormal VOMS testing	Activities permitted at home: - Slow walking for short durations, stopping if symptoms increase by >2 points on 0–10 VAS [50] - Daily tasks and chores (dishes, meal prep, light cleaning) - Movements that can be performed with little effort (do not increase breathing and/or heart rate significantly) - Student moves to Step 2 when able to complete activity described above with no more than “mild” (no greater than 2-point increase) and “brief” (<1 h) symptoms and no new symptoms	Physical therapy assessment may include: - Patient-reported outcome measures, including but not limited to DHI, Rivermead Questionnaire, Motion Sensitivity Index, Concussion Clinical Profiles Screen, Convergence Insufficiency Symptom Survey, etc. [85,88,95,96,97,98] - Standardized assessment of balance and/or gaze stability (e.g., FGA, Bess, CTSIB, SOT, Functional Reach Test, PDMS-2, DGI, pediatric balance scale, and/or BOT-2 [98,99,100,101,102,103,104,105,106,107,108,109] - Ocular screen including the following [69]: - Cranial nerves III, IV, and VI - Ocular alignment - Spontaneous and/or gaze-held nystagmus - Head Impulse Test/Head Thrust test [67,69,70,74] - Horizontal and Vertical Head Shake Nystagmus test (fixation removed) [70,74] - Smooth pursuits and saccades [70,74] - Dynamic visual acuity [70,74] - Visual motion sensitivity [70,74] - Optokinetic testing [70,74] - Positional testing as indicated [70,74] - Vestibular–Ocular Motor Screening Tool (VOMS) [110,111] - Near Point of Convergence (MCID 4 cm) [97] Referral considerations: Occupational Therapy and/or optometry. Referral to a PT with expertise in vestibular and oculomotor rehabilitation if the evaluating therapist does not have the appropriate expertise in the area
2	May be symptomatic or asymptomatic at rest, symptomatic with activity Potential student presentation: - Ongoing visual disturbance (blurred/double vision, difficulty reading, motion sensitivity) - Dizziness - Oscillopsia - Abnormal smooth pursuits or saccades - VOR dysfunction - Near Point of Convergence >6 cm	Activities permitted in PE class: - Participating in a progressive walking program or stationary bike propulsion in a quiet environment (if possible) o Initial duration of ≤15 min and progressing daily until this workload can be maintained for 15 min without rest and with no more than “mild” (no greater than 2-point increase) and “brief” (<1 h) symptoms and no new symptoms o Use of stationary bike at first may be more appropriate in this domain due to increased sensorimotor demands on treadmill [54,91] - Light-intensity yoga and/or stretching when sitting or standing without inversion	Physical therapy treatment may include: - Exercise prescription based on impairments to create individualized treatment plans following rehabilitation protocols as indicated (positional testing and treatment, adaptation, habituation, and/or substitution) [10,38,57,70,71,72,73,74,75,76,77] - **Canalith repositioning:** IF positional testing is positive for BPPV [70,71,73] - **Adaptation:** IF noted VOR dysfunction and/or oscillopsia [38,70,71,72,73,74] - Begin gaze stability training with goal to enhance recovery of VOR - Begin with static exercises to enhance gaze stabilization, and eye motion with no head motion, such as corrective saccades - Once able to tolerate saccades in seated position, progress to include head motion with or without eye motion (VORx1 and VORx2 starting in a seated position with a plain background). Begin at slower speeds (patient-selected at the level of just provoking symptoms—consider 0.5–1 Hz) - **Habituation/Desensitization:** IF needed to treat exaggerated sense of after-motion (visual–vestibular integration impairment) by providing repeated exposure to the provocative stimulus staring with plain backgrounds in quiet environments [70,71,72,77] - May include VOR cancelation in horizontal and vertical planes with a plain background at a speed that just provokes symptoms - Optokinetic exercises: use of optokinetic backgrounds while sitting with progression to standing - Motion sensitivity exercises: gradual exposure to noxious activities (e.g., head movements, ball circles, head circles, etc.) - **Balance training/coordination [68]:** - Initiate static standing balance starting with eyes open on a firm surface and gradually progress to eyes closed - Initiate dynamic gait training as tolerated including walking with horizontal and vertical head turns in a quiet, indoor environment Goals to progress to next step: - Asymptomatic VOR on firm surfaces with plain backgrounds at metronome speeds of 120 bpm and 40 bpm, respectively (6 Hz frequency) - No increase in symptoms with optokinetic exercises
3	Student may be asymptomatic or symptomatic at rest, symptoms do not increase by more than 2 points with activities performed in Step 2 Potential student presentation: - Visual symptoms improving, though continue to exacerbate with fatigue, stimulus, or increased cognitive challenge - Fogginess, balance, and focus improving - Ongoing dizziness, though improving and at decreased frequency	Activities permitted in PE class: - Aerobic exercise: stationary bike, elliptical, treadmill, or flat-surface jogging for progressive intervals with walking “breaks” between o Intervals begin at a 1:1 ratio (jog/rest) - Initiate simple manipulative activities as tolerated (i.e., ball pass/catch, dribbling, etc.) - Individual activities in gym (e.g., throwing drills, shooting drills in basketball and soccer) in predictable and controlled environments with no risk of re-injury - Initiate bodyweight and isotonic weight training with RPE <4/10 on modified Borg [51] o E.g., bodyweight squats and pushups, 1 set of 10 reps each [59]	Physical therapy treatment may include: - **Positional:** Retest if indicated. Perform additional repositioning maneuvers as needed [70,71] - **Gaze stabilization:** Progress VOR demand by increasing head velocity, decreasing the distance to the target, increasing the number of repetitions, progressing to a busy background, and/or switching to a VORx2 paradigm [67,71] - Decrease speed of metronome at first if needed - **Habituation/Desensitization [67,71,72,73,74,75]:** - Optokinetic exercises: use of optokinetic backgrounds in sitting with additional head movements in pitch and yaw plane as tolerated - Motion sensitivity exercises progress to include gait/dynamic activities with head turns - VOR cancelation in the horizontal and vertical planes with busy backgrounds - Further progress to busy environments - **Balance/coordination:** Progress to narrow base of support on compliant surfaces with eyes open or closed [67,71] Goals to progress to next step: - No increase in symptoms with VORx1 on unstable surfaces and/or with busy visual backgrounds - No increase in symptoms with VORx2 on stable surfaces
4	Symptoms improving, occurring less often and at lower intensity, symptoms do not increase with activity in Step 3 Potential student presentation: - May be asymptomatic or symptomatic at rest	Activities permitted in PE class: - Progressively increase physical activity to non-contact training drills to add coordination and increased thinking challenges - Initiate simple locomotor activities (galloping, hopping, leaping, skipping) - Dynamic warmups (high knees, butt kicks, carioca, etc.) - Unit-specific non-contact activity in a SAFE environment modified for individual work - Progress resistance program to circuits (e.g., squat, deadlift, bench press, etc.) - Aerobic exercise within gym environment and/or outdoors	Physical therapy treatment may include: - **Gaze stabilization:** Progress VOR demand in the horizontal and vertical planes by including increased speeds (up to 120 bpm) with a busy or optokinetic background [71,73,74,75] - Add dual-task/divided-attention component to VOR exercises - Progress VORx2 exercises to include a busy background or unstable surfaces - **Habituation/Desensitization:** Progress to include busy environments and backgrounds with increased motor demands (e.g., backward ball toss) [67,71,73,74,75] - **Balance/coordination:** Progress static balance exercises to include head turns (horizontal and vertical) with eyes closed on compliant surfaces [67,71] - Progress gait to include walking with 360 degree turns every 4 steps in a busy environment [71,72,73] Goals to progress to next step: - No increase in symptoms with VORx1 under all conditions (surface progression and background progression) - Tolerating gait activities with head turns without symptom exacerbation
5	No increase in symptoms with activities performed Potential student presentation: - Student demonstrating improved tolerance for vestibular input without symptoms or loss of balance	Activities permitted in PE class: - Unit-specific non-contact activity relevant to curriculum benchmarks in a SAFE environment - Initiate small-group skill work: station work, small side volley games, more intense aerobic activity o Progress gradually to complex training drills with or without a partner (e.g., passing drills in soccer and hockey, post moves and rebounding in basketball, fielding ground balls and throwing/catching in baseball) - Begin participation in practices for non-contact interschool sports Elementary: throw and catch with a partner using safe equipment, rhythm activity, station work MS/HS: - High-resistance weight training with a spotter - Plyometric exercises (burpees, squat thrusts, mountain climbers)	Physical therapy treatment may include: - Complete VORx1 in the horizontal and vertical planes at increased speeds (up to 120bpm) with dynamic balance challenges (e.g., forward and backward walking) [71,72,73] - Progress visual motion habituation to include dynamic motions in busy environments (e.g., backward ball toss with walking forward and backward) - Progress balance interventions to include inversion and rotational movements (e.g., alternating lunges with head/body rotation) - Progress gait to include higher speed of movement with rapid pivots and turns (e.g., jogging with 180-degree pivot turns every 4 steps forward and backward in a busy environment) [71,72] Goals to progress to next step: - No symptoms with VOMS assessment - No symptoms with gaze stabilization activities with dual-task/divided-attention activities - No symptom exacerbation with dynamic movements (e.g., head turns - Clinically relevant improvements on self-reported questionnaires and functional outcome measures tested at baseline
6	Discharge from PT—transition to RTP protocol as needed		The student scores within normal or expected ranges per balance, exertion, and symptom assessment measurements as indicated per the nature of their illness and impairments. Consider completion of return-to-sports assessment (e.g., Dynamic Exertion Test for Concussion) [52,94]

**Table A3:** Autonomic Dysfunction and Exertion Intolerance Guideline.

Step	Status	Return to PE Progression	Physical Therapy Program
1	Symptoms are acute, experiencing symptoms at rest and/or symptom onset immediately with activity Potential student presentation: - Exertional intolerance - Reported symptoms (e.g., dizziness, headache, increased sweating, rapid heart rate) that worsen with activity - Irregularities with temperature - Tension and mood dysfunction - Gastrointestinal dysfunction - Physiologic threshold (increase in symptoms prior to 90% of age-predicted HR max) during BCTT, BCBT, or other form of exertion testing	Activities permitted at home: - Slow walking for short durations, stopping if symptoms increase by >2 points on 0–10 VAS [50] - Daily tasks and chores (dishes, meal prep, light cleaning) - Movements that can be performed with little effort (do not increase breathing and/or heart rate significantly) - Student moves to Step 2 when able to complete activity described above with no more than “mild” (no greater than 2-point increase) and “brief” (<1 h) symptoms and no new symptoms	Physical therapy assessment may include: - Resting vitals - Test for orthostatic hypotension and autonomic dysfunction (resting and postural tachycardia) [10,56,69] - BCTT, Buffalo Concussion Bike Test, McMaster All-Out Progressive Continuous Cycling Test, or similar exertion assessment completed [37,91,112] - Testing modality (treadmill vs. stationary bike) based off symptoms at rest/symptom irritability [54,91] - Patient-reported outcome measures such as Barriers to Being Active Quiz, Rivermead Questionnaire, etc. [85,113] - Exercise prescription based off symptom-limited heart rate threshold (HRt) of BCTT/graded exertional tolerance test, typically prescribed exercise up to 80–90% of HRt, but stopping if symptoms increase by 2–3 points on VAS [10,34,35,36,37,40,54,56,57,58,60] - Adapt the student’s exercise program if positive for POTS to follow established guidelines (e.g., Levine, CHOP) [114] - Referral considerations: - Cardiology and/or neurology - Counseling or psychiatry/neuropsychiatry [115]
2	May be symptomatic or asymptomatic at rest, symptomatic with activity Potential student presentation: - Ongoing autonomic dysregulation, which may worsen with stress, stimulus, or environment challenge	Activities permitted in PE class: - Participating in a progressive walking program or stationary bike propulsion in a quiet environment (if possible) - Initial duration of ≤15 min and progressing daily until this workload can be maintained for 15 min without rest and with no more than “mild” (no greater than 2-point increase) and “brief” (<1 h) symptoms and no new symptoms - Light-intensity yoga and/or stretching when sitting or standing without inversion	Physical therapy treatment may include: - Gradual, progressive aerobic exercise - Consider starting in supine or reclined positions such as rowing or recumbent cycling - Mindfulness-based interventions, vagus nerve calming strategies, and diaphragmatic breathing techniques for symptom management [116,117] - Counterpressure measures (e.g., isometric holds) [116,117] - Strengthening interventions in a seated or supine position - Emphasize lower extremity and core as indicated by strength assessment Goals to progress to next step: - Nearing ability to achieve 80% of HRt based off exertion testing with ≤2-point increase in symptoms (utilizing preferred mode: treadmill, stationary bike, etc.) [10,34,35,36,37,40,54,56,57,58,60]
3	Student may be asymptomatic or symptomatic at rest, symptoms do not increase by more than 2 points with activities performed in Step 2 Potential student presentation: - Continued autonomic dysregulation, though some symptoms may be resolved or diminished	Activities permitted in PE class: - Aerobic exercise: stationary bike, elliptical (stationary arms), treadmill, or flat-surface jogging for progressive intervals with walking “breaks” between - Initiate simple manipulative activities as tolerated (i.e., ball pass/catch, dribbling, etc.) - Individual activities in gym (e.g., throwing drills, shooting drills in basketball and soccer) in predictable and controlled environments with no risk of re-injury - Initiate bodyweight and isotonic weight training (e.g., bodyweight squats and pushups, 1 set of 10 reps each) [59]	Physical therapy treatment may include: - Progress duration and intensity of aerobic exercise challenge in upright positions (e.g., elliptical, treadmill, etc.) - Progress strengthening interventions for resistance and repetitions to target muscle groups Goals to progress to next step: - Student able to tolerate up to 85% of age-predicted max HR on BCTT or similar exertion test with no increase in symptoms [10,34,35,36,37,40,54,56,57,58,60]
4	Symptoms improving, occurring less often and at lower intensity, symptoms do not increase with activity in Step 3 Potential student presentation: - May be asymptomatic or symptomatic at rest	Activities permitted in PE class: - Progressively increase physical activity to non-contact training drills to add coordination and increased thinking challenges - Initiate simple locomotor activities (galloping, hopping, leaping, skipping) - Dynamic warmups (high knees, butt kicks, carioca, etc.) - Unit-specific non-contact activity in a SAFE environment modified for individual work - Progress resistance program to circuits (e.g., squat, deadlift, bench press, etc.) - Aerobic exercise within gym environment and/or outdoors	Physical therapy treatment may include: - Progress duration and intensity of aerobic exercise with multiplanar movement (e.g., vertical displacement with jumping/hopping, and horizontal dynamic movements such as “cutting”) - Progress strengthening interventions for resistance and repetitions to target muscle groups Goals to progress to next step: - Tolerating 90% of age-predicted HR max running on even ground; elliptical; plyometric and agility drills [10,34,35,36,37,40,54,56,57,58,60]
5	No increase in symptoms with activities performed Potential student presentation: - Increased tolerance for activity, positioning, and stimulus	Activities permitted in PE class: - Unit-specific non-contact activity relevant to curriculum benchmarks in a SAFE environment - Initiate small-group skill work: station work, small side volley games, more intense aerobic activity - Progress gradually to complex training drills with or without a partner (e.g., passing drills in soccer and hockey, post moves and rebounding in basketball, fielding ground balls and throwing/catching in baseball) - Participation in practices for non-contact interschool sports Elementary: throw and catch with a partner using safe equipment, rhythm activity, station work MS/HS: - High-resistance weight training with a spotter - Plyometric exercises (burpees, squat thrusts, mountain climbers)	Physical therapy treatment may include: - Progress duration and intensity of aerobic exercise to include rotary and acceleration/deceleration movements - Progress strengthening interventions for resistance and repetitions to target muscle groups Goals to progress to next step: - Tolerating 20–40 min of interval/HIIT training with max HR up to 95% age-predicted HR max and low at 50–60% [10,34,35,36,37,40,54,56,57,58,60] - Tolerating up to 95% of age-predicted HR max running on uneven ground [10,34,35,36,37,40,54,55,56,57,58,60]
6	Discharge from PT—transition to RTP protocol as needed		The student scores within normal or expected ranges per balance, exertion, and symptom assessment measurements as indicated per the nature of their illness and impairments. Consider completion of return-to-sports assessment (e.g., Dynamic Exertion Test for Concussion) [52,94]

**Table A4:** Motor Function Impairment Guideline.

Step	Status	Return to PE Progression	Physical Therapy Program
1	Symptoms are acute, experiencing symptoms at rest and/or symptom onset immediately with activity Potential student presentation: - Student may endorse feeling unsteady when standing or walking - Balance problems - Dizziness - Impaired coordination (e.g., “clumsiness”)	Activities permitted at home: - Slow walking for short durations, stopping if symptoms increase by >2 points on 0–10 VAS [50] - Daily tasks and chores (dishes, meal prep, light cleaning) - Movements that can be performed with little effort (do not increase breathing and/or heart rate significantly) - Student moves to Step 2 when able to complete activity described above with no more than “mild” (no greater than 2-point increase) and “brief” (<1 h) symptoms and no new symptoms	Physical therapy assessment may include: - Patient-reported outcome measures (e.g., Rivermead Questionnaire, Activities-Specific Balance Confidence Scale) [85,118] - Orthostatic vitals assessment [64,69] - Static and dynamic balance [64,69] - Motor coordination tests [64,69] - Dual/multitasking ability with and without balance challenge [69,90] - Computerized posturography if available [70,77] - VOMS to rule in/out vestibuloculomotor impairment [71,73,110,111] - Dual-task/divided-attention assessment (e.g., dual-task during gait, cognitive TUG) [119,120] - Standardized assessment of balance and/or gross motor skills (e.g., Bess, CTSIB, SOT, PDMS-2, and/or BOT-2) [101,104,105,106,107,108,109,121,122] - Exertion assessment on treadmill or bike as appropriate [36,37,91]
2	May be symptomatic or asymptomatic at rest, symptomatic with activity Potential student presentation: - Improving postural stability in controlled environments without increased cognitive demand - Decreased muscle activation and force production - Impaired motor task performance with or without a simultaneous cognitive task	Activities permitted in PE class: - Participating in a progressive walking program or stationary bike propulsion in a quiet environment (if possible) o Initial duration of ≤15 min and progressing daily until this workload can be maintained for 15 min without rest and with no more than “mild” (no greater than 2-point increase) and “brief” (<1 h) symptoms and no new symptoms - Light-intensity yoga and/or stretching when sitting or standing without inversion - Static standing balance exercises (e.g., feet together or heel–toe with eyes open/closed for 30 s trials)	Physical therapy treatment may include: - Maintained holds of single-task static balance at an appropriate level of difficulty as determined by balance testing on FIRM surfaces - Initiate dual-task/divided-attention activities as tolerated on FIRM surfaces in controlled environments [10,72,73] - Static balance with head movements as appropriate [72,73] Goals to progress to next step: - Student able to maintain static balance on FIRM surfaces without symptom exacerbation for appropriate durations as determined by age- and gender-matched norms - Student performing divided-attention tasks on FIRM surfaces with ≤2/10 increase in symptoms
3	Student may be asymptomatic or symptomatic at rest, symptoms do not increase by more than 2 points with activities performed in Step 2 Potential student presentation: - Improving postural stability with increasing environmental stimulus - Improving control with muscle activation and force production with familiar tasks	Activities permitted in PE class: - Aerobic exercise: stationary bike, elliptical, treadmill, or flat-surface jogging for progressive intervals with walking “breaks” between - Initiate simple manipulative activities as tolerated (i.e., ball pass/catch, dribbling, etc.) - Individual activities in gym (e.g., throwing drills, shooting drills in basketball and soccer) in predictable and controlled environments with no risk of re-injury - Dynamic balance activities as tolerated - E.g., tandem walking forwards and backwards, ball throws to self or a partner while maintaining balance positions, balance with increasing stimulus or cognitive demand - Initiate bodyweight and isotonic weight training (e.g., bodyweight squats and pushups, 1 set of 10 reps each) [59]	Physical therapy treatment may include: - Progress balance exercises on FIRM surfaces to include dynamic skills and movements [10,57,67,68,69,70,71,72,73] - May initiate static standing balance exercises on COMPLIANT surfaces [10,57,67,68,69,70,71,72,73] - Progress dual-task activities as tolerated [10,57,67,68,69,70,71,72,73] Goals to progress to next step: - Student able to maintain dynamic balance on FIRM surfaces without symptom exacerbation - Student able to maintain static balance on COMPLIANT surfaces without symptom exacerbation for appropriate durations - MCID improvement on balance assessments (e.g., BOT-2 balance subtest, pediatric Berg, Bess balance, SOT, CTSIB-M, etc.) [101,104,107,108,109,121,122]
4	Symptoms improving, occurring less often and at lower intensity, symptoms do not increase with activity in Step 3 Potential student presentation: - May be asymptomatic or symptomatic at rest - Improving postural stability with increasing environmental stimulus and cognitive demand - Improving control with muscle activation and force production with familiar and novel tasks	Activities permitted in PE class: - Progressively increase physical activity to non-contact training drills to add coordination and increased thinking challenges - Initiate simple locomotor activities (galloping, hopping, leaping, skipping) - Dynamic warmups (high knees, butt kicks, carioca, etc.) - Unit-specific non-contact activity in a SAFE environment modified for individual work - Progress resistance program to circuits (e.g., squat, deadlift, bench press, etc.) - Aerobic exercise within gym environment and/or outdoors	Physical therapy treatment may include: - Dynamic balance interventions with interval dual-task or divided-attention activities [57,72,73] - Progress cognitive challenge as appropriate - Progress dynamic balance exercises to COMPLIANT surfaces [57,72,73] Goals to progress to next step: - No symptom exacerbation with dynamic movements on FIRM or COMPLIANT surfaces - No symptom exacerbation, increased time, deterioration of form, or cues required with dual-task or divided-attention activities
5	No increase in symptoms with activities performed Potential student presentation: - Motor control and postural stability significantly improved in both calm and busy environments	Activities permitted in PE class: - Unit-specific non-contact activity relevant to curriculum benchmarks in a SAFE environment - Initiate small-group skill work: station work, small side volley games, more intense aerobic activity - Progress gradually to complex training drills with or without a partner (e.g., passing drills in soccer and hockey, post moves and rebounding in basketball, fielding ground balls and throwing/catching in baseball) - Participation in practices for non-contact interschool sports Elementary: throw and catch with a partner using safe equipment, rhythm activity, station work MS/HS: - High-resistance weight training with a spotter - Plyometric exercises (burpees, squat thrusts, mountain climbers)	Physical therapy treatment may include: - Integrate sport-specific movements and activities into balance exercises Goals to progress to next step: - Static and dynamic balance meeting age- and gender-matched norms OR pre-injury functional level per appropriate outcome measures - Tolerating dual-task activities without symptom exacerbation - Tolerating balance activities with visual and/or vestibular demands in neck torsion without symptom exacerbation
6	Discharge from PT—transition to RTP protocol as needed		The student scores within normal or expected ranges per balance, exertion, and symptom assessment measurements as indicated per the nature of their illness and impairments. Consider completion of return-to-sports assessment (e.g., Dynamic Exertion Test for Concussion) [52,94]
