# Supplementary material for: The Unconsidered Pathway: Suggestions for Physical Therapists to Facilitate Student Reintegration to Physical Education after a Concussion
Source: Children (Basel). 2024 Sep 30;11(10):1206. doi: 10.3390/children11101206 (PMC11506483; doi:10.3390/children11101206)
Supplement: Supplementary file 1 [file children-11-01206-s001.zip › children-3199167-supplementary.pdf]

Patient:____

DOB:____

Diagnosis:______________________________________

Date:____

School:____

Activity Level

Physical Education: Category specified indicates activities allowed

☐ No Physical Education allowed: PT/OT HEP exercises allowed
☐Light Activity: Stretching, walking, stationary cycling, elliptical, or therapy exercises/HEP (No resistance training or ball play.)
☐ Moderate Activity: jogging, brisk walking, body weight resistive training, non-contact (☐No weights)
☐ Full Activity: May participate in all Physical Education Activities with NO RESTRICTIONS
☐ Student is permitted to perform Home Exercise Program provided only during P.E.
☐ Stop activity if symptoms increase
☐ Resume gentle activity if symptoms return to baseline after 5 minute
☐ Stop activities for the day if symptoms continue greater than 5 minutes
☐ Other: ____

☐Return to Competitive Activity or Sport Play-No Restrictions

Restrictions apply until:____

School Accommodations/Special Needs/Restrictions

☐ Allow student to stop or modify activity if complains of pain/fatigue/dizziness/eye strain or other visual difficulties
☐ Homework: None☐ Limited☐ (#of minutes per day________)
☐ Allow extra time to complete assignments
☐ Tests: No tests☐ Limited ☐(#of tests per week________)
☐ Allow student to leave class early to avoid large crowds
☐ Allow student extra time to get to class
☐ Allow student to wear sunglasses, computer glasses
☐ Allow student to wear headphones or earplugs
☐ Allow student to take noise breaks

☐ Allow preferred seating in classroom
☐Front ☐Center ☐ Back
☐ Allow student to use elevator
☐ Allow student to rest in school clinic as necessary
☐ Provide a copy of class notes or board work
☐Print notes on colored paper
☐ Allow other choices for computer work (oral answers only, typewriter, etc.)
☐ Screen time limited to __________minutes/day
☐ Allow student to use a colored overlay for reading and computer work
☐ Allow student to have a quiet place instead of:
☐Music ☐Cafeteria ☐______________
☐Other:____

Accommodations/restrictions apply until: _______________________________

Signatures:

Signature:____ Title: ____
Printed Name: ____ Date:____
